# Predictors of Length of Stay in Hospital After Transcatheter Aortic Valve Replacement: Impact of Naples Prognostic Score

**DOI:** 10.3390/medicina61091658

**Published:** 2025-09-12

**Authors:** Faruk Boyacı, Murat Akcay, Mustafa Kursat Sahin, Mustafa Yenercag, Ahmet Karagoz, Ahmet Yanik, Serkan Sivri, Rustem Yilmaz, Berkant Ozturk, Halil Ibrahim Kokcu, Enes Kaya, Okan Oguzhan Ovaz, Abdalrahman Shawky Abdalrahman Mostafa, Emre Kasim Yilmaz

**Affiliations:** 1 Department of Cardiology, Samsun Education and Research Hospital, Samsun University, 55090 Samsun, Turkey; faruk_0601@hotmail.com (F.B.); mustafayenercag@hotmail.com (M.Y.); drahmetkgz@hotmail.com (A.K.); drserkansivri@gmail.com (S.S.); drrustemyilmaz@hotmail.com (R.Y.); berkant52.dr@gmail.com (B.O.); halilkokcu314@gmail.com (H.I.K.); dreneskaya@gmail.com (E.K.); okanovaz67@hotmail.com (O.O.O.); drabdurrahmanlacin@gmail.com (A.S.A.M.); emreyilmaz25374@gmail.com (E.K.Y.); 2 Department of Cardiology, Faculty of Medicine, Ondokuz Mayıs University, 55139 Samsun, Turkey; drmuratakcay@hotmail.com; 3 Department of Family Medicine, Faculty of Medicine, Ondokuz Mayıs University, 55139 Samsun, Turkey; 4 Clinic of Cardiology, Samsun Medicina Hospital, 55080 Samsun, Turkey; dr_ahmetynk@hotmail.com

**Keywords:** length of hospital stay, naples prognostic score, transcatheter aortic valve replacement (TAVR)

## Abstract

*Background and Objectives:* Preoperative systemic inflammation and nutritional status are known to affect prognosis and length of hospital stay (LoS) in patients undergoing transcatheter aortic valve replacement (TAVR). The Naples Prognostic Score (NPS) is a simple and effective scoring system that assesses both nutrition and inflammation, and has been shown to predict prognosis in various clinical settings. We aimed to determine the effect of NPS on LoS in patients undergoing TAVR. *Materials and Methods:* A total of 405 patients who underwent TAVR were retrospectively divided into two groups based on length of stay: early discharge (LoS ≤ 3 days) and late discharge (LoS > 3 days). The NPS was calculated prior to the TAVR procedure. *Results:* In the late discharge group (*n* = 164, 40.6%), patients were found to have significantly higher values for age, NYHA functional class, STS risk score, systolic pulmonary artery pressure, white blood cell count, neutrophil count, monocyte count, creatinine, glucose, NLR, rate of surgical access site, incidence of major/minor access site and structural complications, myocardial infarction, cerebrovascular events, acute kidney injury, major bleeding, blood transfusion, pacemaker implantation, and elevated NPS (*p* < 0.05). When independent risk factors for late discharge were evaluated, in addition to reduced eGFR, surgical closure of the access site, history of cerebrovascular events, need for pacemaker implantation, and blood transfusion, a high Naples Prognostic Score was also identified as an independent risk factor for prolonged hospital stay after TAVR in the multivariate logistic regression analysis (*p* < 0.05). *Conclusions:* A high Naples Prognostic Score (NPS), which reflects systemic inflammation and nutritional status, is associated with delayed hospital discharge and is an independent risk factor in patients undergoing TAVR.

## 1. Introduction

Aortic stenosis (AS) is a common valvular heart disease in elderly adults and, if left untreated, is associated with high morbidity and mortality. Transcatheter aortic valve replacement (TAVR) has revolutionized the treatment of patients with severe acquired AS. Although initially developed for patients deemed inoperable or at high surgical risk, the use of TAVR continues to expand, and current guidelines support its application in intermediate- and selected low-risk patients as well [1,2]. Despite being a minimally invasive treatment, TAVR candidates often have a high burden of comorbidities, which increases the risk of mortality and/or adverse events. Post-procedurally, these patients often require intensive care monitoring, leading to prolonged hospital stays. A shorter length of hospital stay after TAVR is associated with better clinical outcomes [3,4]. Early discharge after TAVR is important not only for medical reasons but also for healthcare resource planning and cost-effective utilization.

Several risk scoring systems exist to predict outcomes following surgical aortic valve replacement (SAVR) and TAVR. The Society of Thoracic Surgeons (STS) score and EuroSCORE II are routinely used to assess whether patients are suitable for surgical or percutaneous intervention and to estimate in-hospital and 30-day mortality risk [5,6]. However, these traditional scores do not account for factors such as frailty, systemic inflammation, and nutritional status. Pre-procedural assessment of frailty can help predict patients’ recovery after TAVR. Current guidelines recommend assessing frailty in patients prior to undergoing TAVR [1,2]. Serum albumin level reflects nutritional status and is a key component of frailty. A low serum albumin level is a strong predictor of adverse outcomes in patients undergoing TAVR [7,8,9]. Like nutrition, inflammation also plays a significant role in frailty, and systemic inflammation has been shown to be associated with increased mortality and complication rates after TAVR [10,11].

The Naples Prognostic Score (NPS) is a novel scoring system that evaluates patients’ nutritional and inflammatory status. Derived from biochemical markers including serum albumin, total cholesterol, lymphocyte-to-monocyte ratio (LMR), and neutrophil-to-lymphocyte ratio (NLR), NPS was first used to predict prognosis in patients undergoing surgery for colorectal malignancy [12]. Subsequent studies have shown that NPS is also associated with poor outcomes in certain cardiovascular diseases [13,14]. It has also been demonstrated that NPS may be a simple and effective tool for predicting both short- and long-term prognosis in patients undergoing TAVR [15,16]. In this study, we aimed to determine whether pre-procedural NPS also has an effect on hospital length of stay (LoS) in patients undergoing TAVR.

## 2. Materials and Methods

### 2.1. Study Design and Population

In this study, patients with symptomatic severe aortic stenosis who underwent TAVR at the Department of Cardiology of Samsun Training and Research Hospital between December 2017 and June 2022 were retrospectively analyzed. The decision to perform TAVR was made by a multidisciplinary heart team consisting of at least two cardiologists, a cardiovascular surgeon, and an anesthesiologist. Patient data were retrospectively collected from the hospital’s electronic medical records system.

**Figure 1:**
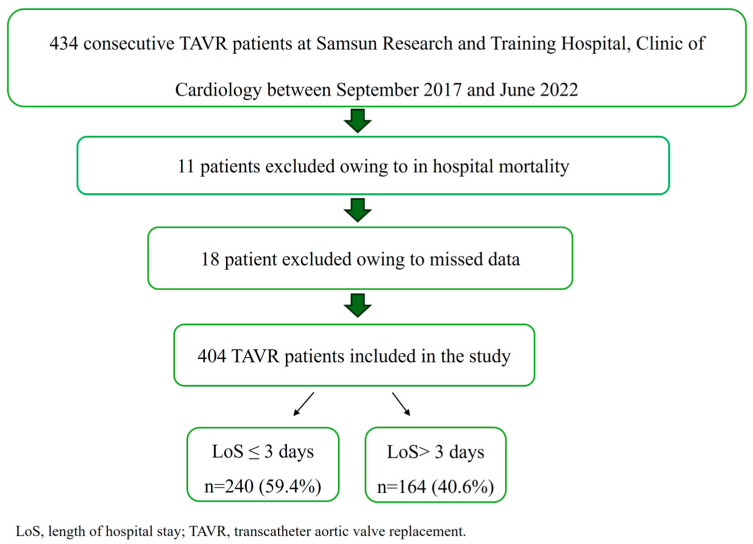
Flowchart of the study population.

Patients with acute infections, hematologic disorders, end-stage liver or renal failure, active immunologic diseases, active malignancies, or those receiving ongoing oncological treatments were excluded from the study. After applying the exclusion criteria, 434 patients were eligible for analysis. Eleven patients who died during hospitalization and eighteen patients with incomplete data were also excluded, leaving a final study population of 404 patients for analysis. The study population was divided into two groups based on the length of hospital stay: early discharge (≤3 days) and late discharge (>3 days) (Figure 1).

Data on patients’ medical history, risk factors, medication use, clinical and demographic characteristics, hematologic and biochemical parameters, echocardiographic measurements, procedural details, procedure-related complications, and STS scores were retrieved from the hospital database. Laboratory values obtained within 24–48 h prior to the TAVR procedure were used for biochemical assessments. The vital status of all patients was verified through the Turkish National Death Index. This study was conducted in accordance with the principles of the Declaration of Helsinki and was approved by the Non-Invasive Clinical Research Ethics Committee of Samsun University (GOKAEK 27 December 2025).

### 2.2. Periprocedural Imaging and TAVR Procedure

To assess the severity of AS, transthoracic echocardiographic imaging was performed prior to the TAVR procedure. Computed tomography (CT) scans were conducted to evaluate the anatomy of the ascending aortic wall and the aortic valve. The TAVR procedures were performed by the same operators in a standard cardiac catheterization laboratory. Different valves [balloon expandable: Edwards Sapien XT, Edwards Sapien 3 (Edwards Lifesciences, Irvine, CA, USA) and Myval Transcatheter Heart Valves (Meril Life Sciences Pvt. Ltd., Vapi, Gujarat, India); self-expandable: Medtronic Corevalve (Medtronic Inc., Minneapolis, MN, USA)] were applied depending on the operator’s preference. All patients underwent the procedure via the transfemoral approach. All procedures were carried out under conscious sedation.

Length of stay in the intensive care unit (ICU) and overall hospital length of stay were recorded. The decision to discharge was made based on medical assessment by the institution’s local team and according to standard local practice. Procedural complications such as vascular complications, bleeding, red blood cell transfusion requirements, acute kidney injury, permanent pacemaker implantation, and new cerebrovascular events were retrieved from the digital database.

### 2.3. Definitions

Total length of stay was calculated from the day of the TAVR procedure (Day 0) until the day of discharge. ICU stay was defined as the number of days from the procedure to the point when the patient was transferred to the general ward. Early discharge was defined as discharge ≤ 3 days after TAVR, while late discharge was defined as discharge > 3 days after the procedure (4). All complications related to the TAVR procedure including myocardial infarction, cerebrovascular events, acute kidney injury, vascular or bleeding complications were recorded [17]. The cutoff of 3 days for early discharge was selected based on previous literature and evolving institutional practices aiming for minimalist TAVR protocols [3,4,7,8,15]. This threshold is also pragmatically aligned with the median LoS (3 days) in our study and reflects a clinically meaningful distinction in healthcare resource utilization.

### 2.4. Assessment of the Naples Prognostic Score

**Figure 2:**
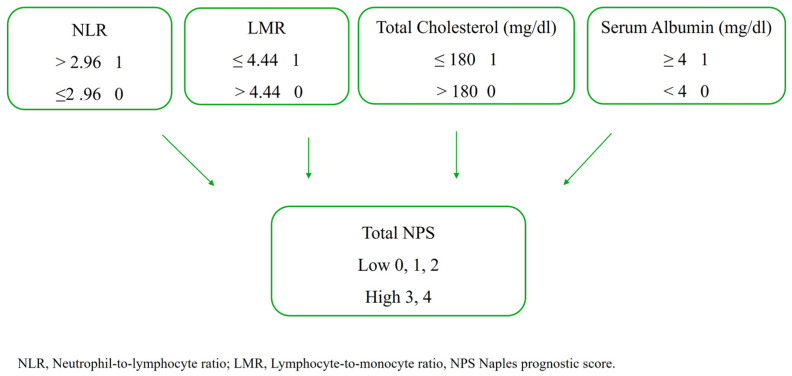
The calculation of the NPS.

Baseline routine laboratory parameters and those specific to the Naples Prognostic Score (NPS) were recorded based on values obtained 24–48 h prior to the TAVR procedure. The NPS is calculated using serum albumin and total cholesterol concentrations, lymphocyte-to-monocyte ratio, and neutrophil-to-lymphocyte ratio. The NPS was computed according to a previously described method. Following NPS calculation, patients were categorized into two groups: the low NPS group (NPS 0–2) and the high NPS group (NPS 3–4) [14] (Figure 2).

### 2.5. Statistical Analysis

All statistical analyses were performed using IBM SPSS Statistics for Windows, version 25.0 (IBM Corp., Armonk, NY, USA). The normality of distribution for continuous variables was assessed using the Kolmogorov–Smirnov test. Since the majority of continuous variables were not normally distributed, they are presented as median and interquartile range (IQR). Comparisons of these variables between the two groups (early vs. late discharge; low vs. high NPS) were performed using the non-parametric Mann-Whitney U test. Categorical variables were expressed as frequency (*n*) and percentage (%), and differences between groups were assessed using Pearson’s chi-square test or Fisher’s exact test where appropriate (e.g., when expected cell counts were less than 5).

To identify independent predictors of early discharge (≤3 days), univariate logistic regression analyses were first conducted for all variables. Variables found to be statistically significant in univariate analyses were included as candidates for the multivariate logistic regression model. To mitigate the risk of multicollinearity, variance inflation factors (VIF) were calculated for all candidate variables. Variables with a VIF > 5 were considered to indicate significant multicollinearity and were carefully evaluated; however, no variables exceeded this threshold, supporting their inclusion as independent predictors. The model was further tested for potential interactions between the included variables, particularly between NPS and other strong predictors such as eGFR and surgical access site closure. The Hosmer-Lemeshow test indicated a good fit of the final model to the observed data (χ^2^(8) = 7.261, *p* = 0.509). No significant interaction terms were identified that improved the model’s fit or interpretability based on the likelihood ratio test. The final model was thus constructed using the backward elimination method to include only the main effects of the significant predictors. For each variable in the final model, the odds ratio (OR) and 95% confidence interval (CI) were calculated. The performance of the final multivariate model was assessed using Cox & Snell R^2^, Nagelkerke R^2^, and overall classification accuracy.

Additionally, subgroup analyses were performed by classifying patients into low and high NPS risk groups. The associations between NPS level and length of hospital stay, post-procedural complications, and laboratory parameters were evaluated. A two-tailed *p*-value of <0.05 was considered statistically significant.

## 3. Results

Of the 404 patients included in the study, 240 (59.4%) were discharged early, while 164 (40.6%) were discharged late. In the late discharge group, patients were significantly older and had higher NYHA functional class, STS risk scores, systolic pulmonary artery pressure, white blood cell count, neutrophil count, monocyte count, creatinine, glucose, NLR, and a higher incidence of surgical access site use. Additionally, rates of major and minor access site and structural complications, myocardial infarction, cerebrovascular events, acute kidney injury, major bleeding, blood transfusion, and pacemaker implantation were significantly higher in this group. Conversely, levels of hemoglobin, lymphocytes, glomerular filtration rate (GFR), total protein, albumin, total cholesterol, NMR, and left ventricular ejection fraction were significantly lower in the late discharge group. Notably, patients with a high Naples Prognostic Score (NPS) were significantly more likely to be in the late discharge group (Table 1).

When patients were stratified according to the Naples Prognostic Score, 212 patients (52.5%) were classified into the low NPS group, while 192 patients (47.5%) fell into the high NPS group. Patients with a high NPS were significantly older, had higher STS risk scores, worse NYHA class, and a higher prevalence of diabetes, hyperlipidemia, and coronary artery disease. They also had higher systolic pulmonary artery pressure, increased rates of major and minor complications, cerebrovascular events, myocardial infarction, acute kidney injury, surgical closure of the access site, bleeding, blood transfusion, pacemaker implantation, and longer ICU and total hospital stays (Table 2).

Independent risk factors associated with prolonged hospital stay were analyzed using univariate and multivariate logistic regression analyses (Table 3). In addition to reduced eGFR, surgical closure of the access site, history of cerebrovascular events, pacemaker implantation, and need for blood transfusion, a high NPS was identified as an independent predictor of prolonged hospital stay following TAVR in the multivariate logistic regression analysis (Table 3).

## 4. Discussion

This single-center, retrospective study demonstrated that a high preprocedural NPS is an independent predictor of prolonged hospital stay (LoS > 3 days) in patients undergoing TAVR. To the best of our knowledge, this is the first study to specifically evaluate this relationship.

Most TAVR candidates present with a high burden of comorbidities and an increased risk of mortality and/or adverse events. Despite advances in TAVR techniques and technologies, predicting outcomes and identifying patients at elevated risk of postprocedural complications remains a significant clinical challenge. Identifying reliable predictors is crucial for optimizing patient selection, achieving better outcomes, and enabling discharge at the earliest appropriate time. Innovations in device design and procedural techniques have made TAVR increasingly less invasive and have facilitated the development of a minimalist approach. Early discharge using a minimalist strategy has been shown to be safe, to promote faster recovery, to reduce hospital length of stay, and to lower overall healthcare costs [3]. In recent years, hospital stays for patients undergoing TAVR have significantly shortened, leading to reductions in healthcare expenditures [18,19,20]. Our data revealed a median ICU stay of 1.3 days and a median total LoS of 3.8 days after TAVR. Factors such as advanced age, NYHA class ≥ 3, high STS risk score, reduced ejection fraction, elevated systolic pulmonary artery pressure, impaired preprocedural renal function, lower hemoglobin and albumin levels, high NPS, postprocedural complications, and the need for new permanent pacemaker implantation were found to significantly influence LoS and were associated with delayed discharge.

Frailty is common in patients with severe AS and is associated with worse outcomes [21,22,23]. Current guidelines recommend evaluating frailty prior to TAVR [1,2]. AS is a chronic condition characterized by low-grade inflammation, which can lead to impaired physical performance, loss of appetite, and poor nutritional status. Decreased serum cholesterol and albumin levels, along with reduced lymphocyte counts, are indicative of malnutrition. These markers not only reflect changes in nutritional status but also highlight the extent of systemic inflammation. Several studies have shown that poor nutritional status negatively affects the prognosis of TAVR patients. Well-established nutritional scoring systems such as the Geriatric Nutritional Risk Index (GNRI), Controlling Nutritional Status (CONUT) score, and Prognostic Nutritional Index (PNI) have been studied in the context of TAVR [23,24,25]. While these scores are valuable, a direct comparison of their predictive power for LoS specifically remains underexplored. The NPS incorporates inflammatory ratios (NLR, LMR) in addition to nutritional markers (albumin, cholesterol), potentially offering a more holistic view of the patient’s physiological reserve and stress response. This composite nature might make it particularly adept at predicting recovery-based outcomes like LoS, as inflammation is a key driver of post-procedural complications and delayed healing. Future head-to-head studies comparing NPS with GNRI, CONUT, and PNI for predicting LoS would be valuable to identify the most efficient tool for clinical use. Serum albumin, a common marker of nutritional status, is a key contributor to frailty and a strong predictor of poor outcomes in TAVR patients [7,8,9]. Low serum albumin has also been linked to adverse outcomes in other cardiovascular conditions [26]. Like nutrition, inflammation plays a critical role in frailty, and systemic inflammation has been associated with increased mortality and complication rates after TAVR [10,11]. Although frailty itself was not evaluated in this study, we focused specifically on the relationship between NPS and LoS. Frailty encompasses a broad spectrum of factors and cannot be solely attributed to nutrition and inflammation. Nonetheless, NPS remains a valuable tool for assessing inflammatory and nutritional status, and prior studies have demonstrated its utility in predicting both short- and long-term outcomes in TAVR patients [15,16].

Nutritional status is a significant determinant of prognosis and treatment outcomes in patients with cardiovascular disease [27]. Malnutrition may prolong hospital stay by increasing the risk of complications and reducing treatment efficacy, thereby raising hospitalization costs. Previous studies have shown that nutritional status is linked to LoS in patients admitted for cardiovascular reasons [28,29,30]. Moreover, preprocedural hypoalbuminemia has been associated with longer hospital stays in TAVR patients [31]. Similarly, our study found that patients in the LoS > 3 days group had significantly lower serum albumin levels. Systemic inflammation, like malnutrition, plays a critical role in the pathophysiology of adverse outcomes following TAVR [10,11,32]. So, preprocedural TAVR risk evaluation using scores that evaluate systemic inflammation and malnutrition, such as NPS, and necessary support before the procedure will have a positive impact on long-term clinical outcomes. Novel inflammatory biomarkers such as the NLR and LMR have emerged as potential indicators. In our study, patients with LoS > 3 days had higher NLR and lower LMR levels.

Optimizing LoS after TAVR is not only medically beneficial reducing the risk of hospital-acquired infections and other complications by also contributes to lower procedure-related and resource-utilization costs. In conclusion, NPS is a valuable tool for assessing nutritional and inflammatory status. Our study is novel in that it investigated the impact of preprocedural NPS on hospital stay duration in patients undergoing TAVR. Our findings suggest that high preprocedural NPS is a good predictor of prolonged LoS (>3 days) in this population. Similarly, Gitmez et al. [15] demonstrated in their retrospective evaluation of 222 TAVR patients that NPS may be a potential predictor of long-term mortality and major adverse cardiovascular events (MACE). Hakgor et al. [16], in a study of 343 TAVR patients, also found NPS to be a simple and effective score for predicting both short- and long-term prognosis. Erdoğan et al. [33] reported that in 173 patients undergoing surgical aortic valve replacement (SAVR), NPS was an independent predictor of postoperative mortality. Consistent with our findings, a recent study by Jiritano et al. highlighted that major bleeding is a critical complication in elderly patients undergoing TAVR, occurring in approximately 10% of cases and significantly contributing to adverse outcomes and resource utilization [34]. Our results, showing that major bleeding and transfusion need are independent predictors of prolonged LoS, align perfectly with this evidence. The NPS, by reflecting a state of underlying inflammation and malnutrition, may identify patients with impaired vascular integrity and coagulopathy, thereby acting as a proxy for heightened bleeding risk. This biological plausibility strengthens the link we observed between a high NPS, peri-procedural complications, and ultimately, delayed discharge. In light of these findings, patients with a high NPS may benefit from closer follow-up and targeted interventions aimed at correcting malnutrition and controlling inflammation to help optimize LoS. It is important to note that our results warrant further validation in larger, prospective studies.

A valid question arising from our findings is whether the NPS is merely a surrogate for a higher burden of traditional comorbidities. While our univariate analysis confirmed that patients with a high NPS had a higher prevalence of conditions such as diabetes, hyperlipidemia, and coronary artery disease, the multivariate logistic regression analysis was crucial in addressing this concern. By adjusting for these and other clinically relevant confounders (including STS score, which aggregates several comorbidities), we demonstrated that a high NPS remained a powerful independent predictor of prolonged LoS (OR: 29.756, *p* < 0.001). This suggests that the NPS provides prognostic information above and beyond what is captured by conventional risk scores and comorbidity indices. It likely reflects the integrated pathophysiological impact of systemic inflammation and malnutrition, which can exacerbate a patient’s vulnerability to procedural stress and hinder recovery, irrespective of their underlying diagnostic labels.

The clinical utility of the preprocedural NPS lies in its ability to flag patients at high risk for a complicated and prolonged recovery. Identifying such patients preemptively can refine risk stratification beyond traditional surgical scores. This knowledge could prompt a multi-faceted approach: (1) intensified pre-habilitation efforts focusing on nutritional optimization and medical therapy to modulate inflammatory status, if possible; (2) setting realistic expectations with patients and families regarding the anticipated hospital course; (3) ensuring heightened vigilance in the peri-procedural period for early signs of complications (e.g., bleeding, infection, delirium); and (4) facilitating early involvement of multidisciplinary teams (dietitians, physiotherapists, case managers) to plan for a safe discharge, potentially mitigating avoidable delays. While interventional studies are needed to confirm that acting on a high NPS improves outcomes, its assessment provides a simple, objective, and holistic metric to inform personalized patient management.

Several limitations of this study should be acknowledged. First, the relatively small sample size, single-center design, and retrospective nature, respectively, represent the major limitations that may affect the generalizability of the findings. Second, our study focused exclusively on the relationship between the NPS and hospital LoS, without addressing other components of frailty, which may also influence patient outcomes. Third, while our multivariate model adjusted for a wide array of clinical and procedural variables, the potential for residual confounding remains. Factors not captured in our database, such as socioeconomic status, health literacy, availability of social support at home, and institutional variations in peri-discharge care and rehabilitation protocols, could also significantly influence the decision to discharge a patient and thus affect LoS. These unmeasured confounders represent an inherent limitation of our retrospective study design. Additionally, due to the dynamic nature of inflammatory and nutritional biomarkers, serial assessments rather than single time-point measurements could provide deeper and more accurate prognostic insights. A further limitation is the absence of a direct comparison between the NPS and other nutritional/inflammatory scores (e.g., CONUT, GNRI, PNI, CRP) in predicting LoS. Future studies designed to concurrently calculate and compare the prognostic utility of these various scores are warranted to determine if the NPS offers superior predictive value in the TAVR population. Future prospective, multicenter studies with larger cohorts and longitudinal biomarker evaluations are warranted to confirm and expand upon these findings.

## 5. Conclusions

A high NPS, which reflects systemic inflammation and nutritional status, is an independent risk factor associated with delayed hospital discharge in patients undergoing TAVR. When assessed routinely before the procedure, a high NPS may not only help predict prolonged length of stay but also contribute to evaluating the risk of morbidity and mortality. These findings highlight the potential clinical value of incorporating NPS into the preprocedural assessment of TAVR candidates. Further validation through prospective, multicenter studies with larger patient populations is recommended to support and expand upon the current evidence.

## Figures and Tables

**Table 1:** Baseline demographic and clinical characteristics of the patients.

Variables	LoS ≤ 3 Days *n =* 240 (59.4%) Median [IQR] or *n* (%)	LoS > 3 Days *n =* 164 (40.6%) Median [IQR] or *n* (%)	*p*-Values *
**Baseline characteristics**			
Age (years)	79 [75.5–82.5]	80.5 **[77–83]**	0.008
Sex (male)	115 (60.5)	75 (39.5)	0.666
BMI (kg/m^2^)	24.6 [22–29.2]	24.3 [22.1–29.4]	0.915
NYHA (≥3)	126 (48.3)	135 (51.7)	<0.001
Hypertension	156 (57.1)	117 (42.9)	0.181
Diabetes mellitus	75 (48.7)	79 (51.3)	0.001
Hyperlipidemia	106 (62.4)	64 (37.6)	0.304
Peripheral artery disease	28 (56.0)	22 (44.0)	0.600
Cerebrovascular disease	8 (47.1)	9 (52.9)	0.290
Coronary artery disease	87 (52.7)	78 (47.3)	0.023
Prior CABG	35 (64.8)	19 (35.2)	0.385
Atrial fibrillation	49 (57.6)	36 (42.4)	0.710
COPD	85 (61.6)	53 (38.4)	0.519
Smoking	20 (55.6)	16 (44.4)	0.622
STS risk score	7.5 [6.4–8.8]	10.2 [8.5–12.1]	<0.001
**Laboratory parameters**			
WBC (×1000/µL)	7.0 [6–8.3]	7.6 [6.3–9.1]	0.010
Hemoglobin (g/dL)	12.2 [11.6–13.2]	11.2 [10.6–11.9]	<0.001
Lymphocyte (×1000/µL)	1.7 [1.4–2.1]	1.3 [1–1.7]	<0.001
Neutrophil (×1000/µL)	4.4 [3.7–5.2]	5.8 [4.6–6.8]	<0.001
Monocyte (×1000/µL)	0.5 [0.4–0.7]	0.6 [0.5–0.7]	<0.001
Platelet count (×1000/dL)	229 [185–263]	229 [190–287]	0.372
Creatinine (mg/dL)	0.9 [0.8–1]	1.1 [0.9–1.3]	<0.001
eGFR (ml/min/1.73 m^2^)	70 [63.2–80]	59.3 [46.7–65.3]	<0.001
Total protein (g/dL)	7 [6.7–7.3]	6.9 [6.4–7.2]	0.016
Glucose (mg/dL)	104.5 [94–134]	116.5 [99–154]	0.004
LDL cholesterol (mg/dL)	112.1 [91–140]	104 [91.6–125.6]	0.062
HDL cholesterol (mg/dL)	44.5 [38.5–52]	43 [39–48]	0.137
Total cholesterol (mg/dL)	189 [166.5–216.5]	175.5 [156–204.5]	0.015
NLR	2.6 [2–3.1]	4.2 [3.1–6.1]	<0.001
LMR	3.2 [2.4–4.2]	2 [1.6–2.9]	<0.001
Albumin (g/dL)	4.1 [3.8–4.2]	3.7 [3.5–3.8]	<0.001
**Naples prognostic score**			
− Low NPS (0–2)	193 (91.0)	19 (9.0)	<0.001
− High NPS (3–4)	47 (24.5)	145 (75.5)	
**Echocardiographic parameters**			
Peak aortic gradient (mmHg)	80 [70–90]	77 [69–88]	0.068
Mean aortic gradient (mmHg)	48 [43–55]	47 [42–54.5]	0.140
Aortic valve area (cm^2^)	0.8 [0.7–0.9]	0.8 [0.7–0.9]	0.096
Left ventricular ejection fraction (%)	55 [50–60]	50 [40–55]	<0.001
Systolic pulmonary arterial pressure (mmHg)	38 [35–41.5]	44 [39–50]	<0.001
**Procedural features**			
Valve size (mm)	29 [26–29]	27.5 [26–29]	0.318
Transcatheter Heart Valve Type			
− Balloon Expandable	168 (57.7)	123 (42.3)	0.272
− Self-Expandable	72 (63.7)	41 (36.3)	
Pre-Dilatation	157 (59.5)	107 (40.5)	0.971
Post-Dilatation	27 (54.0)	23 (46.0)	0.406
Access Site Closure			
− Percutaneous	228 (70.6)	95 (29.4)	<0.001
− Surgical	12 (14.8)	69 (85.2)	
Major vascular, access-related or cardiac structural complication	0	8 (100)	0.001
Minör vascular, access-related or cardiac structural complication	6 (16.7)	30 (83.3)	<0.001
Myocardial infarction	4 (16.0)	21 (84.0)	<0.001
Cerebrovascular event	3 (16.7)	15 (83.3)	<0.001
Acute kidney injury	0	13 (100)	<0.001
Pacemaker implantation	9 (33.3)	18 (66.7)	0.004
Major bleeding	3 (14.3)	18 (85.7)	<0.001
Transfusion	16 (25.8)	46 (74.2)	<0.001

* Chi-square test, Mann-Whitney U test.

**Table 2:** Demographic and clinical variables of patients with low and high NPS.

Variables	Low NPS (0–2) *n =* 212 (52.5%) Median [IQR] or *n* (%)	High NPS (3–4) *n =* 192 (47.5%) Median [IQR] or *n* (%)	*p*-Values *
Age (years)	78 [75–82]	81 [78–84]	<0.001
Sex (male)	100 (52.6)	90 (47.4)	0.953
BMI (kg/m^2^)	24.8 [22–29.3]	24.3 [22–29.2]	0.773
NYHA (≥3)	108 (41.4)	153 (58.6)	<0.001
Hypertension	139 (50.9)	134 (49.1)	0.365
Diabetes mellitus	69 (44.8)	85 (55.2)	0.015
Hyperlipidemia	100 (58.8)	70 (41.2)	0.029
Peripheral artery disease	22 (44.0)	28 (56.0)	0.200
Cerebrovascular disease	7 (41.2)	10 (58.8)	0.341
Coronary artery disease	73 (44.2)	92 (55.8)	0.006
Prior CABG	27 (50.0)	27 (50.0)	0.696
Atrial fibrillation	41 (48.2)	44 (51.8)	0.378
COPD	76 (55.1)	62 (44.9)	0.451
Smoking	20 (55.6)	16 (44.4)	0.698
STS risk score	7.5 [6.0–8.7]	9.9 [8.0–11.9]	<0.001
Peak aortic gradient (mmHg)	80 [70–90]	78 [70–88]	0.106
Mean aortic gradient (mmHg)	48 [43–55]	48 [42–55]	0.499
Aortic valve area (cm^2^)	0.8 [0.7–0.9]	0.8 [0.7–0.9]	0.760
Left ventricular ejection fraction (%)	55 [50–60]	50 [40–60]	0.001
Systolic pulmonary arterial pressure (mmHg)	38 [35–40.5]	42 [38–48]	<0.001
Valve size (mm)	28 [26–29]	29 [26–29]	0.942
Transcatheter heart valve type			
− Balloon expandable	150 (51.5)	141 (48.5)	0.549
− Self-expandable	62 (54.9)	51 (45.1)	
Pre-dilatation	141 (53.4)	123 (46.6)	0.606
Post-dilatation	26 (52.0)	24 (48.0)	0.943
Access site closure			
− Percutaneous	195 (60.4)	128 (39.6)	<0.001
− Surgical	17 (21.0)	64 (79.0)	
Major vascular complication	3 (37.5)	5 (62.5)	0.392
Minor vascular complication	9 (25.0)	27 (75.0)	0.001
Myocardial infarction	4 (16.0)	21 (84.0)	<0.001
Cerebrovascular event	4 (22.2)	14 (77.8)	0.009
Acute kidney injury	2 (15.4)	11 (84.6)	0.006
Pacemaker implantation	9 (33.3)	18 (66.7)	0.039
Major bleeding	5 (23.8)	16 (76.2)	0.007
Transfusion	22 (35.5)	40 (64.5)	0.004
Intensive care unit stay (day)	1 [1–1]	1 [1–2]	<0.001
Total hospital stays (day)	3 [2–3]	4 [4–5]	<0.001

* Chi-square test, Mann-Whitney U test.

**Table 3:** Univariate and multivariate logistic regression analysis of independent predictors of LoS > 3 days.

Variables	Univariate OR (95% CI)	*p* Value	Multivariate * OR (95% CI)	*p* Value
Age (years)	1.057 (1.014–1.103)	0.009		
NYHA (≥3)	4.212 (2.621–6.769)	<0.001		
Diabetes mellitus	2.045 (1.357–3.082)	0.001		
Coronary artery disease	1.595 (1.065–2.389)	0.023		
STS risk score	1.696 (1.508–1.907)	<0.001		
WBC (×1000/µL)	1.114 (1.022–1.214)	0.014		
Hemoglobin (g/dL)	0.394 (0.313–0.497)	<0.001		
Lymphocyte (×1000/µL)	0.276 (0.183–0.417)	<0.001		
Neutrophil (×1000/µL)	1.653 (1.429–1.911)	<0.001		
Monocyte (×1000/µL)	11.625 (3.961–34.112)	<0.001		
Creatinine (mg/dL)	82.432 (27.486–247.215)	<0.001		
eGFR (ml/min/1.73 m^2^)	0.913 (0.894–0.932)	<0.001	0.936 (0.910–0.964)	<0.001
Glucose (mg/dL)	1.006 (1.002–1.010)	0.003		
Total cholesterol (mg/dL)	0.994 (0.989–0.999)	0.035		
NLR	1.520 (1.348–1.715)	<0.001		
LMR	0.419 (0.336–0.524)	<0.001		
Albumin (g/dL)	0.02 (0.008–0.048)	<0.001		
Naples prognostic score (high NPS)	31.338 (17.639–55.677)	<0.001	29.756 (14.071–62.922)	<0.001
Left ventricular ejection fraction (%)	0.951 (0.930–0.973)	<0.001		
Systolic pulmonary arterial pressure (mmHg)	1.134 (1.096–1.174)	<0.001		
Access site closure (surgical)	13.800 (7.146–26.649)	<0.001	8.518 (3.346–21.683)	<0.001
Minor vascular complication	8.731 (3.543–21.516)	<0.001		
Myocardial infarction	8.664 (2.915–25.750)	<0.001		
Cerebrovascular event	7.953 (2.264–27.937)	0.001	7.071 (1.356–36.875)	0.020
Pacemaker implantation	3.164 (1.385–7.232)	0.006	4.974 (1.276–19.394)	0.021
Major bleeding	9.740 (2.820–33.641)	<0.001		
Transfusion	5.458 (2.962–10.054)	<0.001	4.746 (1.787–12.609)	0.002

* Cox & Snell R Square = 0.531; Nagelkerke R Square = 0.717; Accuracy = 85.4%.

## Data Availability

The original contributions presented in this study are included in the article. Further inquiries can be directed to the corresponding authors.
